# Feasibility of quantitative pulmonary function imaging in real-world cardiovascular magnetic resonance

**DOI:** 10.1007/s10554-026-03702-z

**Published:** 2026-04-10

**Authors:** Jan Gröschel, Josephine Hausmann, Leonhard Grassow, Richard Hickstein, Philine Reisdorf, Edyta Blaszczyk, Phillip van Dijck, Jasmin Zernikow, Robert Grimm, Andreas Voskrebenzev, Jens Vogel-Claussen, Jeanette Schulz-Menger

**Affiliations:** 1 Charité – Universitätsmedizin Berlin, corporate member of Freie Universität Berlin and Humboldt-Universität zu Berlin, Berlin, Germany; 2 Working Group on Cardiovascular Magnetic Resonance, Experimental and Clinical Research Center, Charité Medical Faculty, Delbrueck Center for Molecular Medicine, Department of Cardiology and Nephrology, HELIOS Hospital Berlin-Buch, Medical University Berlin, Charité Campus Buch, Lindenberger Weg 80, 13125 Berlin, Germany; 3 DZHK (German Centre for Cardiovascular Research), Partner site Berlin, Berlin, Germany; 4 Department of Cardiology, Angiology and Intensive Care Medicine, Deutsches Herzzentrum der Charité, Charitéplatz 1, 10117 Berlin, Germany; 5 Research & Clinical Translation, Magnetic Resonance, Siemens Healthineers AG, Erlangen, Germany; 6 Institute of Diagnostic and Interventional Radiology, Hannover Medical School, Hannover, Germany; 7 Biomedical Research in Endstage and Obstructive Lung Disease Hannover (BREATH), Member of the German Center for Lung Research, Hannover, Germany; 8 Department of Radiology, Charité Universitätsmedizin Berlin, Corporate member of Freie Universität Berlin and Humboldt-Universität zu Berlin, Berlin, Germany; 9 Department of Cardiology and Nephrology, HELIOS Hospital Berlin-Buch, Berlin, Germany

**Keywords:** Cardiovascular magnetic resonance, Pulmonary assessment, PREFUL, Feasibility

## Abstract

Providing insights into the feasibility of pulmonary function assessment in real-world cardiovascular magnetic resonance (CMR) practice by applying Phase-REsolved FUnctional Lung imaging (PREFUL). We retrospectively analyzed consecutive patients who underwent PREFUL imaging in addition to routine 1.5T CMR between September 2023 and January 2024. PREFUL was acquired in three coronal slices, with a prototype tool used to derive quantitative perfusion and ventilation defect percentages (QDP and VDP, respectively). Cardiac function was assessed from short-axis cine images. Subgroup analyses included patients with primary pulmonary disease and reduced left ventricular ejection fraction (LVEF). Statistical analyses comprised linear regression, correlation analysis, and Kruskal-Wallis test. The final cohort included N = 172 patients (74 females), median age 60 years (IQR 46–71). PREFUL was feasible in all cases (mean scan time 60 s/slice). Multivariable regression with bootstrap-based backward selection showed associations of QDP with LVEF, pulmonary disease, age, and BMI (all p ≤ 0.005), while VDP was associated with pulmonary disease, age, and male sex (all p < 0.001). QDP correlated negatively with LV stroke volume (ρ (Spearman’s rho) − 0.336, p < 0.001) and cardiac output (ρ − 0.360; p < 0.001) and was higher in patients with LVEF < 50% (p < 0.001). Patients with primary pulmonary disease had higher QDP (p = 0.005) and VDP (p = 0.036). No correlations between cardiac function and VDP were detected (all p > 0.05). Application of PREFUL is feasible and fast in CMR routine. While QDP and VDP are affected by pulmonary disease, QDP is additionally associated with markers of cardiac function and was higher in patients with reduced LVEF.

**Graphical Abstract:**

**Figure:**
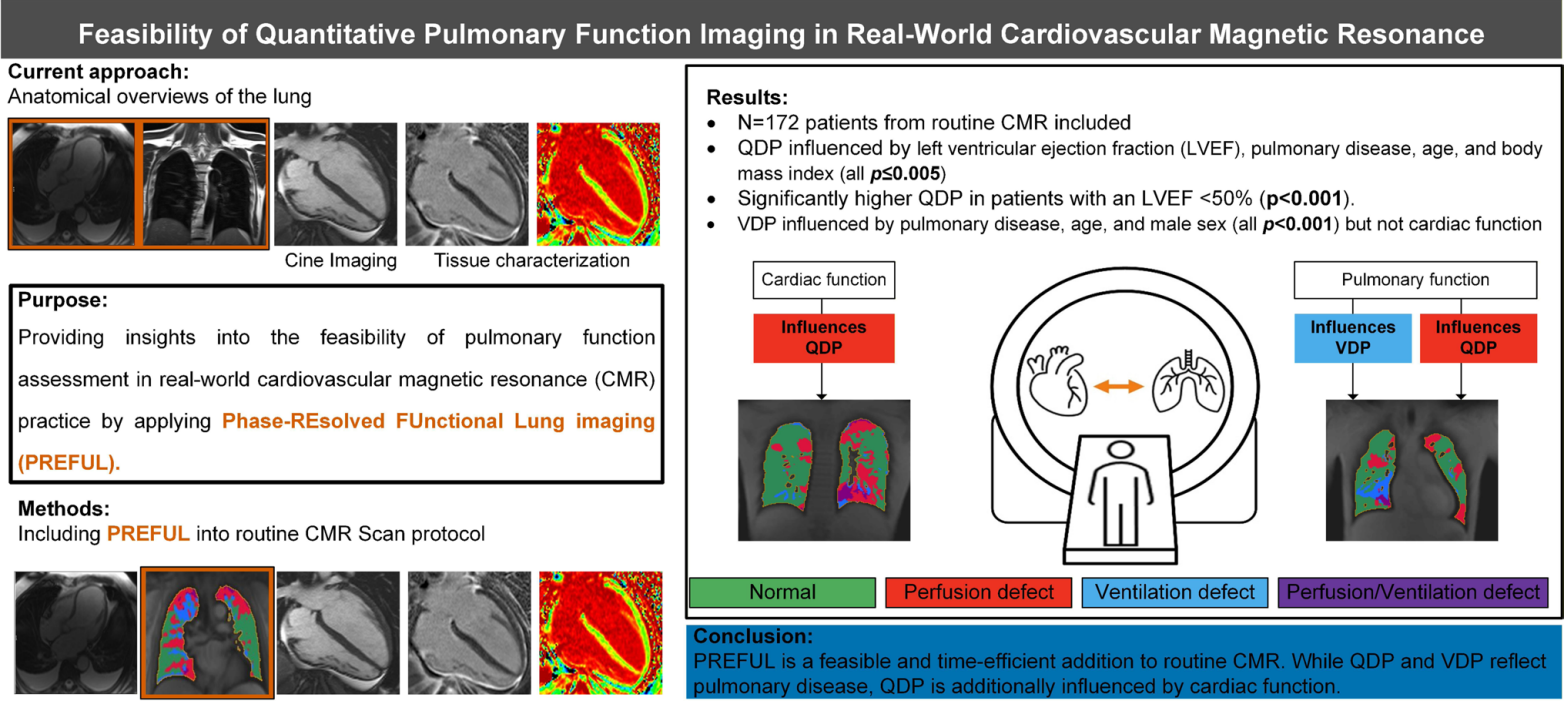
Using the Phase-REsolved FUnctional Lung imaging (PREFUL) sequence is feasible and fast as a complementary addition to routine clinical cardiovascular magnetic resonance (CMR), without a significant time investment.

## Introduction

Patients with heart failure (HF) often suffer from pulmonary diseases, and vice versa [1, 2]. Deciphering the leading cause of a patient’s complaints is often challenging, due to a nonspecific clinical presentation with dyspnea, chest pain, cyanosis or peripheral edema. This overlap is also evident on a pathophysiological level as patients with HF and a reduced left ventricular (LV) function as well as patients with pulmonary disease show increased pulmonary capillary pressures [3, 4]. To accurately diagnose patients presenting with dyspnea, a combined cardiopulmonary assessment would be beneficial. Cardiac function and cardiovascular pathologies can be evaluated accurately and diagnosed in routine cardiovascular magnetic resonance (CMR) [5, 6]. In contrast, only limited options exist for a functional evaluation of the lung through magnetic resonance imaging (MRI), based on physiological properties of the lung. While sequences providing an anatomical assessment [7] have been implemented, many pathologies can only be detected through the analysis of lung perfusion and ventilation. However, such functional imaging is not performed in routine CMR [1]. The Phase-REsolved FUnctional Lung (PREFUL) MRI technique allows for a contrast-free assessment of pulmonary perfusion and ventilation in free breathing [8, 9]. The PREFUL technique has already been validated in studies, demonstrating a strong correlation between the results for the ventilation analysis and the direct regional visualization of ventilated volume with inhalation-based hyperpolarized ^129^Xe MRI [10, 11]. Similar findings have been observed for perfusion data when compared to reference methods like dynamic contrast-enhanced MRI [12–14].

Combining CMR with PREFUL functional lung imaging enables assessment of cardiopulmonary interactions in a free-breathing, contrast-free manner without reducing patient comfort. This study evaluates these interactions and investigates the feasibility of implementing PREFUL in high-volume clinical CMR.

## Materials and methods

### Study design

We conducted a retrospective study including consecutive patients with a clinical indication to undergo CMR who received an extended scan protocol including the PREFUL sequence. Patients scanned between September 2023 and January 2024 on a 1.5T scanner (Siemens Healthineers, AvantoFIT, Forchheim, Germany) were screened. Inclusion criteria were age > 18 years, available PREFUL image acquisition, and cine imaging in a short-axis stack covering the left and right ventricle (RV). Basic cohort characteristics such as age, height, weight, blood pressure and heart rate were acquired prior to the scan. Comorbidities were derived from the electronic patient record.

### MRI techniques

PREFUL MRI was performed with a fast low angle shot sequence with the following parameters: repetition time: 2.1 ms, echo time: 0.93 ms, field of view: 500 × 500 mm^2^, matrix: 128 × 128 (interpolated to 256 × 256), slice thickness: 15 mm, flip angle: 5°, parallel imaging acceleration factor: 2, temporal resolution: <300 ms/frame, number of measurements: 250. Total acquisition duration was 60 s per slice.

All cine images were based on balanced steady state free precession sequences with retrospective ECG gating. The short axis had the following scan parameters: repetition time: 2.78–3.31 ms, number of reconstructed phases: 30, echo time: 1.19–1.44 ms, field of view: 340–380 × 276–380 mm^2^, matrix: 192 × 156, voxel size: 1.8-2.0 × 1.8-2.0 mm^2^, slice thickness: 7 mm with no gap, flip angle: 74–80°, parallel imaging acceleration factor: 2.

### PREFUL analysis

**Fig. 1:**
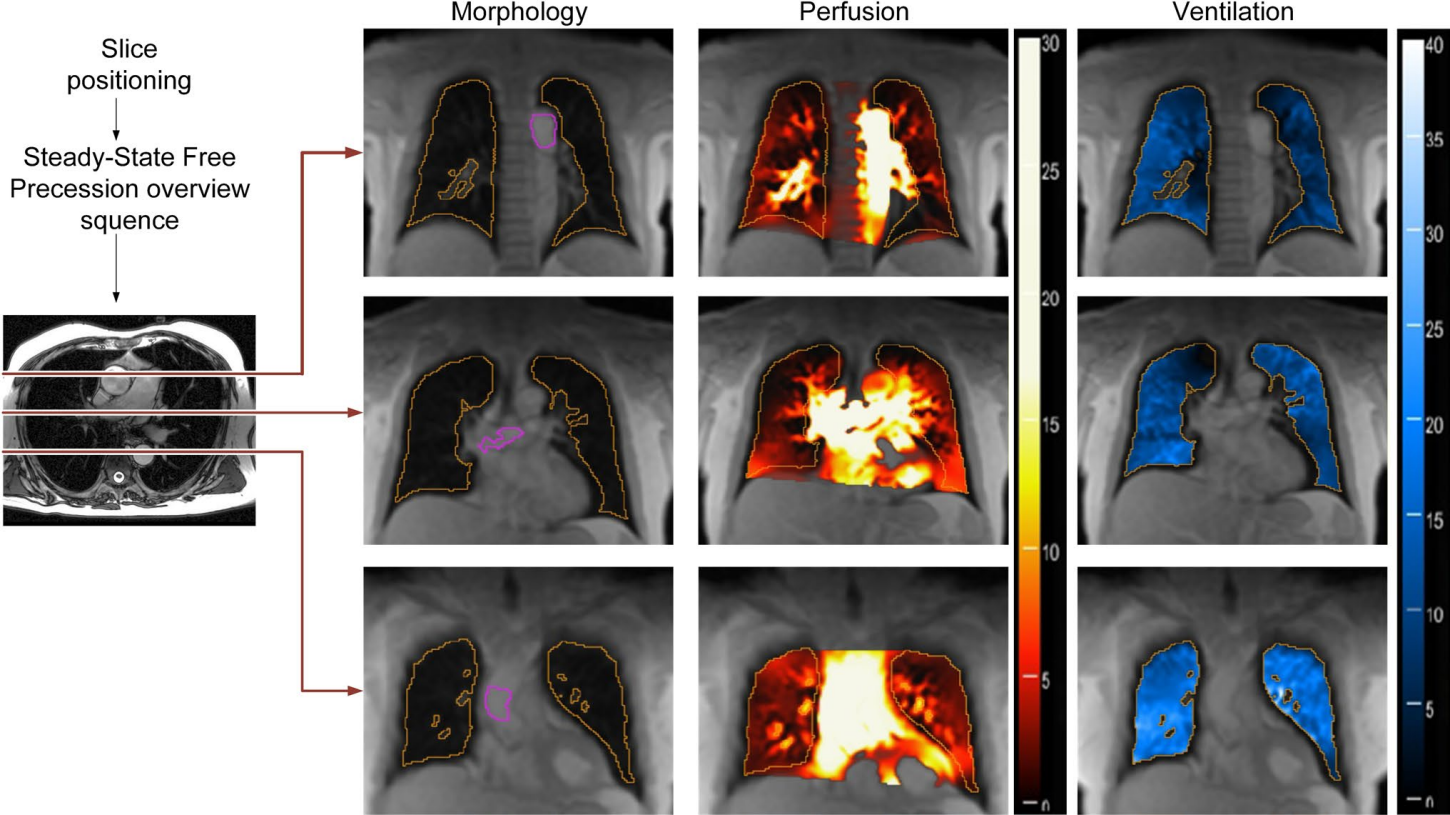
Slice planning for the PREFUL sequence. Slice planning for the PREFUL sequence (left) with the tracheal bifurcation used as the first reference acquisition and with adjacent posterior and anterior slices (maps shown on the right) for representative sampling of the whole lung

Images were acquired in three slices with the middle one being positioned on the bifurcation of the pulmonary artery on standard localizer images (Fig. 1). Post-processing was carried out with a research software tool provided by the vendor (MR Lung v2.3.0; Siemens Healthineers, Forchheim, Germany). Details were published previously [15]. Briefly, ventilation- and perfusion-related signal changes were separated following image registration to an intermediate lung volume by applying low- and high-pass temporal filtering. After estimation of respiratory and cardiac phases, synthesized cycles with increased temporal resolution were reconstructed. Expiratory and inspiratory phases were used to compute regional ventilation (RVent) maps in ml/ml. Cardiac phase information related to parenchymal perfusion was obtained by histogram analysis and used to derive normalized perfusion (QN, expressed as percent) using the signal intensity of a full-blood voxel (e.g., aortic signal) as reference. For further analysis, a convolutional neural network was used to segment the lung parenchyma as the region of interest. Perfusion defects were identified using a fixed threshold of QN < 2.0%, while ventilation defects were determined using an adaptive threshold defined as 0.4 × 90th percentile of the RVent values within the lung parenchyma. The final output included total perfusion and ventilation defect percentages (QDP and VDP, respectively) for each slice as well as a weighted average of all three slices. A weighted mean across slices was calculated based on the lung area covered by each slice. Reproducibility parameters for the PREFUL sequence have been reported previously [16].

### CMR image analysis

All image analyses were carried out by one reader (J.H.) with three years of CMR experience supervised by a second reader (J.G.) with 6 years of CMR experience. Post-processing for cardiac function was carried out using dedicated software (CVI42, version 5.13.7, Circle Cardiovascular Imaging, Calgary, Canada). Biventricular function and volume assessment were carried out in the short-axis cine stack. Endo- and epicardial contours were drawn for the LV in the end-diastolic phase, while only endocardial contours were drawn in end-systole. Papillary muscles were separately contoured in end-systole and end-diastole and included in the overall LV mass. The RV was contoured in end-diastole and end-systole (endocardial contours). All segmentations were carried out according to current recommendations [17].

### Feasibility

To evaluate the feasibility of the PREFUL approach in clinical routine, we assessed both acquisition success and the achievement of diagnostic image quality. Additionally, during post-processing, we examined the accuracy of automatic segmentations and determined whether the quantitative outputs were interpretable.

### Statistical analysis

Data is represented as median and interquartile range (IQR) for continuous variables and as number and percent for categorical variables. Normal distribution was assessed with the Shapiro-Wilk test. Continuous variables were compared globally with the Kruskal-Wallis test and in case of significance followed by pairwise comparisons with the Mann-Whitney U test with Bonferroni correction for multiple testing. Categorical variables were compared with the Chi-square test or Fisher’s exact test. Correlation analysis was carried out using Spearman’s correlation coefficient. In addition, model selection was performed by stepwise ordinary least squares regression with backward elimination of age, sex, body mass index (BMI), LV ejection fraction (LVEF), pulmonary disease (present/absent) as predictors for total QDP and total VDP using an R^2^ criterion. To correct for bias introduced by backward selection we performed model evaluation using a nonparametric bootstrap approach: Backward selection was repeated on 500 bootstrapped samples of the dataset, and for each variable retained in the final models, the mean coefficient, standard deviation, 95% confidence intervals, and inclusion frequency were calculated to provide a robust estimate of model parameters and prevent overfitting. Two-tailed empirical *p*-values were calculated at a level of *p* < 0.05. A *p* < 0.05 was defined as a statistically significant difference. Statistical tests were performed using IBM SPSS Statistics (version 29.0.0.0.) and Python 3.12.11, using the pandas 2.2, statsmodels 0.14.4 and scikit-learn 1.7.0 packages.

## Results

### General characteristics

The final cohort consisted of *N* = 172 patients (74 females, 98 males) with a median age of 60 years (IQR 46–71), weight 78 kg (70–90), height 173 cm (165–180) and BMI 26.2 kg/m^2^ (23.4–29.7). Systolic and diastolic blood pressures were 122 mmHg (112–131) and 67 mmHg (61–75), respectively. Median heart rate was 69 beats/minute (63–79). Main indication for the clinical scans was suspected coronary artery disease (92/172; 53.5%), followed by myocarditis (39/172; 22.7%), primary cardiomyopathy and valvular diseases. Diagnosis was confirmed in 48/172 cases (27.9%). Common comorbidities included arterial hypertension (94/172; 54.7%), hyperlipidemia (48/172; 27.9%) and coronary artery disease (62/172; 36.0%). Details can be found in Table 1.

**Table 1:** Summary of the cohort characteristics

Parameter	Entire Cohort (*N* = 172)
Demographics and Vital signs	
Age (years)	60 (46–71)
Height (cm)	173 (165–180)
Weight (kg)	78 (70–90)
BMI (kg/m^2^)	26.2 (23.4–29.7)
Systolic blood pressure (mmHg)	122 (112–131)
Diastolic blood pressure (mmHg)	67 (61–75)
Heart rate (beats/minute)	69 (63–79)
Scan indication	
Coronary artery disease	92/172 (53.5%)
Myocarditis	39/172 (22.7%)
Cardiomyopathy	24/172 (14.0%)
Valvular disease	13/172 (7.6%)
Others	4/172 (2.3%)
Comorbidities	
Arterial hypertension	94/172 (54.7%)
Diabetes mellitus	28/172 (16.3%)
Chronic heart failure	22/172 (12.8%)
Hyperlipidemia	48/172 (27.9%)
Coronary artery disease	62/172 (36.0%)
Peripheral artery disease	6/172 (3.5%)
Atrial fibrillation	21/172 (12.2%)
Valvular heart disease	47/172 (27.3%)
Chronic kidney disease	20/172 (11.6%)
Pulmonary disease	35/172 (20.3%)

Note: IQR= interquartile range, BMI= body mass index

### Cardiopulmonary function analysis

The functional and volumetric analysis of the ventricles revealed the following median (IQR) values: LV end-diastolic volume 134 ml (118–164), LV stroke volume (SV) 85 ml (75–99), LVEF 63% (57–68), cardiac output (CO) 6.3 L/min (5.1–7.4), LV mass 93 g (74–113), RV end-diastolic volume 142 ml (120–167), RV stroke volume 70 ml (60–85) and RV ejection fraction (RVEF) 50% (46–55).

**Fig. 2:**
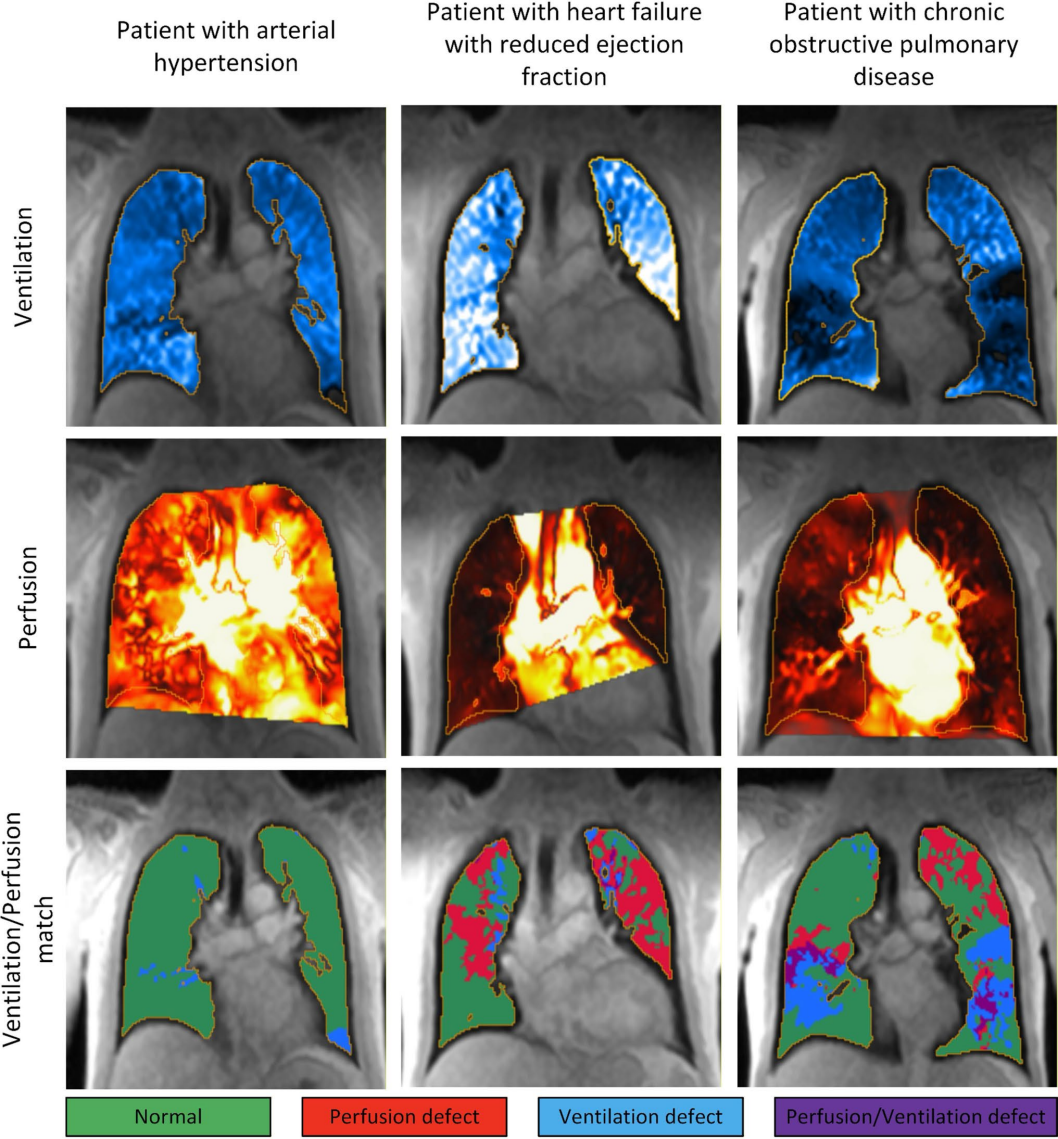
Exemplary images of pathologies for three patients. Exemplary images of pathologies for three patients with respective regional ventilation (V) in ml/ml, normalized perfusion (Q) in percent, and V/Q match maps. V/Q match illustrates regions with isolated ventilation and perfusion defects (blue / red) as well as matched regions of defects (purple) and normal lung function (green)

PREFUL analysis was feasible in all cases with all acquisitions yielding diagnostic image quality, with accurate segmentations and interpretable quantitative data. Median (IQR) post-processing time was 72 s (61–83). Median (IQR) values were for QDP 0.9% (0.1–4.5) and for VDP 12.0% (6.6–22.8). Figure 2 provides example images of pathologies.

Significant correlations were found between QDP and biventricular function parameters including LV SV (ρ -0.336; *p* < 0.001), CO (ρ -0.360; *p* < 0.001) and RV SV (ρ -0.302; *p* < 0.001). Neither LVEF (ρ -0.105; *p* = 0.105) nor RVEF (ρ 0.009; *p* = 0.908) showed a significant correlation with QDP. No significant correlations were found between VDP and biventricular function.

Regression analysis revealed QDP to be significantly associated with age (mean standard deviation (0.14 ± 0.03; *p* < 0.001), BMI (-0.31 ± 0.11; *p* = 0.005), LVEF (-0.16 ± 0.05; *p* < 0.001;), and pulmonary diseases (3.2 ± 0.84; *p* < 0.001), while VDP was associated with age (0.19 ± 0.05; *p* < 0.001), male sex (4.4 ± 0.90, *p* < 0.001) and pulmonary diseases (5.90 ± 1.65; *p* = 0.006).

### Subgroup comparisons

#### Subgroup comparisons based on left ventricular ejection fraction

**Fig. 3:**
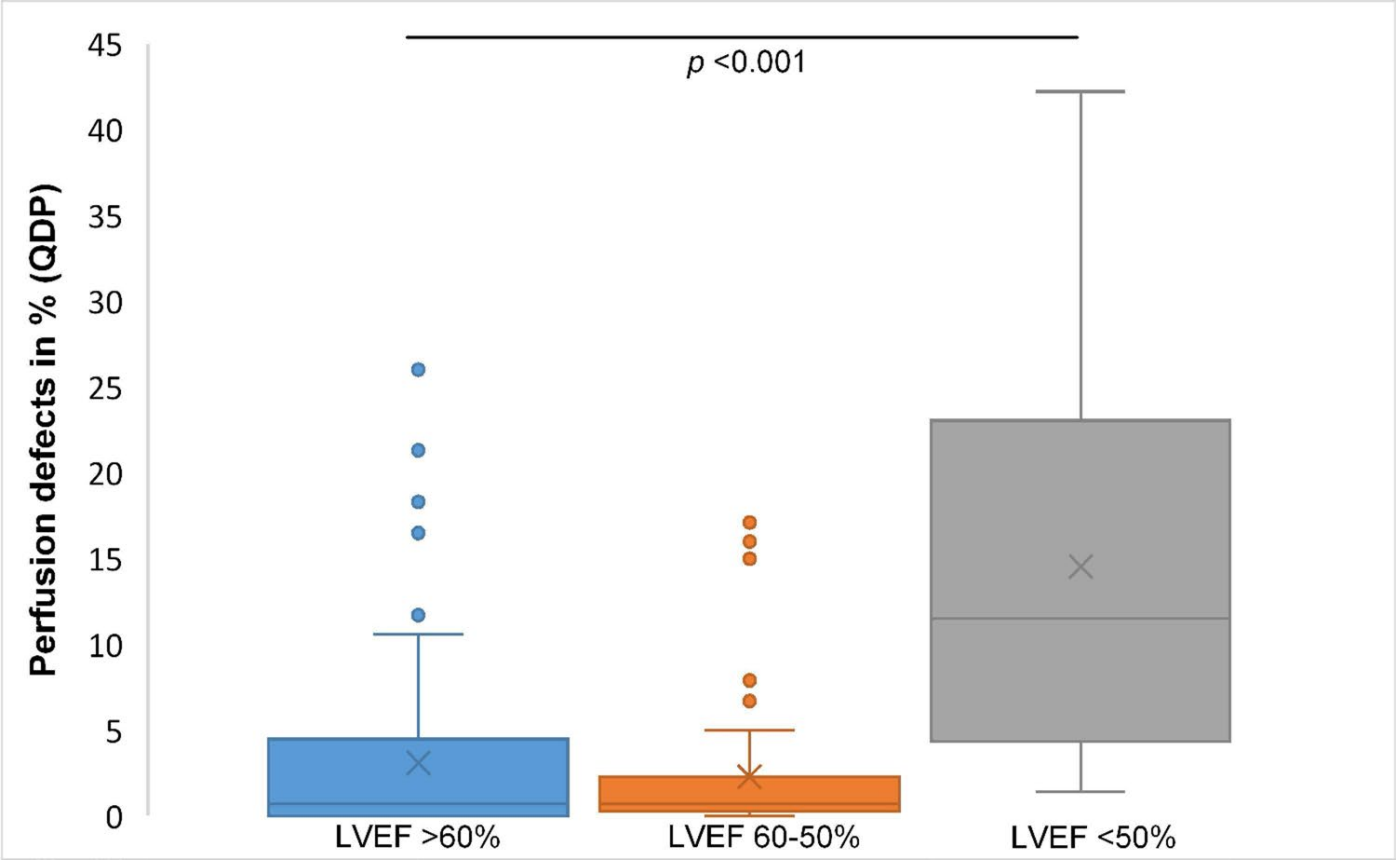
Boxplots for total perfusion defect percentages between the LVEF subgroups. Note: Boxplots representing the median (solid inside the box), interquartile range (box) and 1.5*interquartile range (whiskers) for perfusion defect percentages (QDP). LVEF= left ventricular ejection fraction

To further investigate the effect of LVEF on QDP, we analyzed subgroups divided as follows: *N* = 110 had an LVEF > 60%, *N* = 49 an LVEF 50–60% and *N* = 13 an LVEF < 50% (Table 2). We found significant differences for LVEF (*p* < 0.001) as well as other function/volume parameters between the groups (Table 2). Regarding the pulmonary function analysis, patients with a LVEF < 50% showed higher QDP in comparison to the other two groups (LVEF > 60%: 0.7% (0.0-4.5) vs. LVEF 50–60%: 0.7% (0.25–2.3) vs. LVEF < 50%: 11.5% (4.4–23.1); *p* < 0.001) (Table 2; Fig. 3). We compared the same LVEF subgroups in patients with a RVEF < 40% (*N* = 22). In concordance with the findings regarding higher QDP in patients with a LVEF < 50%, patients with a LVEF < 50% and a RVEF < 40% had higher QDP (*p* = 0.022) in comparison to patients with LVEF of 50–60% and a RVEF < 40% and patients with a LVEF > 60% and a RVEF < 40%.

**Table 2:** Subgroup analysis based on the left ventricular ejection fraction

Parameter	LVEF > 60% (*N* = 110)	LVEF 50–60% (*N* = 49)	LVEF < 50% (*N* = 13)	*p*-value
*Demographics*				
Age (years)	60 (44–70)	60 (47–71)	68 (58–72)	0.376
Height (cm)	173 (166–180)	172 (165–178)	177 (170–182)	0.243
Weight (kg)	79 (70–90)	80 (63–89)	74 (70–78)	0.215
BMI (kg/m	26 (23–30)	26 (24–29)	24 (21–27)	0.071
Systolic blood pressure (mmHg)	120 (113–132)	125 (109–130)	120 (103–133)	0.799
Diastolic blood pressure (mmHg)	67 (61–75)	66 (61–74)	67 (59–78)	0.898
Heart rate (beats/minute)	70 (63–83)	68 (63–78)	67 (65–77)	0.831
*Comorbidities*				
Arterial hypertension	59/110 (54%)	25/49 (51%)	10/13 (77%)	0.234
Chronic heart failure	9/110 (8%)	8/49 (16%)	5/13 (38%)	**0.006** ^**B**^
Coronary artery disease	38/110 (35%)	16/49 (33%)	8/13 (62%)	0.134
Valvular heart disease	25/110 (23%)	14/49 (29%)	8/13 (62%)	**0.012** ^**B, C**^
Diabetes mellitus	18/110 (16%)	6/49 (12%)	4/13 (31%)	0.274
Pulmonary disease	21/110 (19%)	11/49 (22%)	3/13 (23%)	0.861
*Cardiopulmonary function*				
LVEDV (ml)	129 (115–146)	153 (124–171)	184 (124–296)	**< 0.001** ^**A, B**^
LVSV (ml)	86 (77–100)	81 (73–97)	67 (53–103)	**0.037** ^**B**^
LVEF (%)	66 (63–72)	57 (54–58)	42 (36–48)	**< 0.001** ^**A, B,C**^
RVEDV (ml)	141 (119–164)	141 (121–171)	153 (103–205)	0.848
RVSV (ml)	70 (62–83)	70 (55–87)	65 (47–86)	0.780
RVEF (%)	51 (46–55)	49 (46–53)	48 (42–55)	0.188
QDP (%)	0.7 (0.0-4.5)	0.7 (0.25–2.3)	11.5 (4.4–23.1)	**< 0.001** ^**B, C**^
VDP (%)	11.1 (5.8–21.3)	13 (6.8–24.2)	17.2 (11.4–32.1)	0.197

Note: Data represented as median (interquartile range) or as absolute values (percentage). *p* < 0.05 defined as significant. Kruskal-Wallis-Test and Mann-Whitney-U Test with Bonferroni-correction for multiple testing. Significant *p*-values are marked **bold**. ^A^ significant differences between LVEF > 60% and LVEF 60 − 50%, ^B^significant differences between LVEF > 60% and LVEF < 50%, ^C^significant differences between LVEF 60 − 50% and LVEF < 50 LV = Left Ventricle, EDV = end-diastolic volume, SV= stroke volume, EF= ejection fraction, RV= right ventricle, QDP= perfusion deficit percentage, VDP= ventilation deficit percentage

#### Subgroup comparison in patients with pulmonary disease

Of the *N* = 172 patients, *N* = 35 presented with a diagnosis of a pulmonary disease. *N* = 6 with an overlap of asthma and chronic bronchitis, which we included primarily in the asthma subgroup. This cohort included *N* = 10 (10/172; 5.8%) patients with chronic obstructive pulmonary disease, *N* = 18 (18/172; 10.5%) with asthma, *N* = 2 (2/172; 1.2%) with chronic bronchitis, *N* = 3 (3/172; 1.7%,) *N* = 1 (1/172; 0.6%) with pulmonary mass, *N* = 2 (2/172; 1.2%) with pulmonary sarcoidosis and *N* = 1 (1/172; 0.6%) with lung emphysema.

In concordance with the regression analysis, patients with pulmonary disease had higher QDP (pulmonary disease 2.2% (0.6–10.6) vs. no pulmonary disease 0.7% (0.05–3.9); (*p* = 0.005)) and VDP (pulmonary disease 18.8% (8.8–28.8) vs. no pulmonary disease 11.5% (6.5–20.8); *p* = 0.036) (Table 3).

**Table 3:** Subgroup analysis based on pulmonary disease

Parameter	Pulmonary disease (*N* = 35)	No pulmonary disease (*N* = 137)	*p*-value
*Demographics*			
Age (years)	61 (50–72)	60 (45–71)	0.660
Height (cm)	170 (162–176)	174 (168–180)	**0.029**
Weight (kg)	75 (67–85)	80 (70–90)	0.160
BMI (kg/m)	26 (23–31)	26 (24–30)	0.924
Systolic blood pressure (mmHg)	121 (109–129)	123 (112–132)	0.282
Diastolic blood pressure (mmHg)	69 (60–77)	67 (61–75)	0.665
Heart rate (beats/minute)	72 (65–84)	68 (63–78)	0.242
*Comorbidities*			
Arterial hypertension	20/35 (57%)	74/137 (54%)	0.740
Chronic heart failure	8/35 (23%)	14/137 (10%)	**0.046**
Coronary artery disease	12/35 (34%)	50/137 (37%)	0.808
Valvular heart disease	9/35 (26%)	38/137 (28%)	0.811
Diabetes mellitus	10/35 (29%)	18/137 (13%)	**0.027**
*Cardiopulmonary function*			
LVEDV (ml)	124 (102–137)	138 (122–167)	**0.008**
LVSV (ml)	77 (67–85)	87 (77–99)	**0.002**
LVEF (%)	63 (58–70)	62 (57–68)	0.884
RVEDV (ml)	132 (108–154)	144 (121–169)	0.068
RVSV (ml)	65 (53–74)	72 (62–87)	**0.024**
RVEF (%)	51 (45–56)	50 (46–54)	0.950
QDP (%)	2.2 (0.6–10.6)	0.7 (0.05–3.9)	**0.005**
VDP (%)	18.8 (8.8–28.8)	11.5 (6.5–20.8)	**0.036**

Note: Data represented as median (interquartile range) or as absolute values (percentage). *p* < 0.05 defined as significant. Kruskal-Wallis-Test and Mann-Whitney-U Test with Bonferroni-correction for multiple testing. Significant *p*-values are marked **bold**. LV = Left Ventricle, EDV = end-diastolic volume, SV= stroke volume, EF= ejection fraction, RV= right ventricle, QDP= perfusion deficit percentage, VDP= ventilation deficit percentage

## Discussion

In this first study reporting the application of PREFUL MRI during routine CMR scans, we were able to show that functional lung imaging is feasible, fast (~ 3 min), and can help depict how cardiopulmonary diseases influence pulmonary function and perfusion. While pulmonary perfusion and ventilation are primarily influenced by pulmonary diseases, cardiac function parameters, especially in a setting of reduced LVEF, are associated with pulmonary perfusion deficits.

While CMR offers a broad span of sequences for quantitative analysis of myocardial tissue composition and function, the pulmonary system is often only visually analyzed on static anatomical sequences [18]. While these might show pathologies such as pleural effusions or masses, functional lung assessment is not performed routinely. PREFUL MRI has been readily applied in diseases such as chronic obstructive pulmonary disease [9, 19], cystic fibrosis, chronic thromboembolic pulmonary hypertension [12] and post-lung transplantation [20]. In these cohorts, strong correlations were shown between the perfusion and ventilation assessment for PREFUL-derived parameters and the respective gold standards [9, 21].

In this study, we assessed the interaction between the cardiovascular and the pulmonary system with one of the major findings being the association between cardiac function and QDP. A previous study investigating the association between pulmonary perfusion and cardiac function and pressures based on invasive and imaging parameters showed a significant correlation between LVEF and a decreased perfusion slope [22]. However, this finding was not confirmed in a univariate analysis, in which LV end-diastolic pressures in particular were significantly associated with pulmonary perfusion [22]. In CMR, the association between decreased pulmonary perfusion and HF has been linked primarily based on measurements of pulmonary transit time (PTT) [23, 24]. PTT can provide insights into pulmonary perfusion, however, it cannot differentiate between delayed perfusion due to congestion or increased resistance. PREFUL MRI allows for direct visualization of the underlying pathophysiological process, demasking either perfusion or ventilation deficits. It should be noted that we found a correlation between SV and CO with QDP; however, not with LVEF. Potentially this finding might be associated with a majority of patients having no perfusion deficits (0 values) and therefore attenuating a correlation analysis. However, the regression model showed the association between LVEF and QDP. On the one hand, this underlines that cardiac function should be assessed beyond LVEF, and that SV and CO might be better indicators of overall cardiac hemodynamics [25, 26]. On the other hand, this data needs further verification in patients with a reduced LVEF. Note should also be made of the relationship between diastolic function and pulmonary pressures. Especially in patients with HF and preserved function, elevated filling pressures might be the main driver for pulmonary hypertension [27, 28]. Less is known about the HF phenotype with mildly reduced ejection fraction. Further studies investigating this entity and the pathophysiology behind pulmonary hypertension are warranted. While we found an association between cardiac function and QDP, the parameter was also affected by pulmonary disease. A recent study by Duan et al. showed related findings, with increased QDP based on PREFUL MRI in patients with primary pulmonary diseases [29]. Furthermore, the authors provided invasively derived pulmonary pressures, showing increased vascular resistance which might ultimately be the pathophysiologic background for increased perfusion deficits in patients with primary pulmonary diseases.

Increased VDP was not associated with cardiac function. This finding might therefore help to differentiate between cardiovascular or pulmonary diseases as the leading cause of the patient’s symptoms. This finding is relevant as both conditions are frequently encountered together in a patient, with exacerbations of either disease, detrimentally affecting the other [1, 2]. As the underlying pulmonary condition may arise from diverse etiologies, PREFUL has the potential to distinguish between them. A previous study demonstrated high diagnostic performance, with a sensitivity of 97% and a specificity of 95% compared to the gold standard for detecting chronic pulmonary embolism [30]. Further studies are needed to determine whether the PREFUL approach can reliably differentiate between acute and chronic forms.

In summary, the PREFUL technique might help to further decipher the underlying etiology in patients with concomitant cardiopulmonary disease. As a potential outlook, the provided non-invasive markers could aid in diagnosis and therapy guidance [30]. For example, QDP might potentially be used to assess the drug response of patients with reduced LV function, particularly decompensated patients’ response to diuretics. There is evidence that even before diuresis a response in pulmonary vasculature occurs [31, 32].

## Limitations

This is a retrospective, single-center study inherently subject to the limitations of such a design, namely selection bias, incomplete data, and limited generalizability. The limitation of retrospective data is especially evident with regard to standard lung function assessment as well as laboratory biomarkers, such as troponin or NT-proBNP. Further prospective studies integrating these parameters are warranted. In addition, no other imaging modality parameters, such as echocardiography, can be provided. As the study population was based on CMR indications/scans, there is a selection and referral bias. A single-center design also adds to the limitations given that no validation cohort is available. Regarding the scan protocol, only three coronal slices were acquired instead of full-lung coverage, potentially omitting areas of pathology. Lastly, analysis was carried out by one reader without reproducibility testing.

## Conclusion

Application of PREFUL is feasible in CMR routine, providing insights into cardiopulmonary function and interaction, which might be overlooked in the clinical routine in patients with suspected cardiac disease. There is an association between a reduced left ventricular function and increased perfusion deficits, while ventilation defects are mainly associated with pulmonary disease. Further research is warranted on the impact of the PREFUL sequence on differentiating between pulmonary and cardiac causes of dyspnea.

## Data Availability

Data available upon reasonable request and within the German Data Protection Act.

## Abbreviations

HF: heart failure
LV: left ventricle
CMR: cardiovascular magnetic resonance
MRI: magnetic resonance imaging
PREFUL: Phase-REsolved FUnctional Lung
RV: right ventricle
RVent: regional ventilation
QN: normalized perfusion
QDP: perfusion defect percentage
VDP: ventilation defect percentage
IQR: Interquartile range
LVEF: left ventricular ejection fraction
CO: cardiac output
RVEF: right ventricular ejection fraction
PTT: pulmonary transit time

## Declarations

### Consents to participate

Requirements for informed consent to participate in this study were waived due to the retrospective nature of the study by the institutional ethics committee.
